# Microbiome Shapes the T Cell Receptor Repertoire among CD4+CD8+ Thymocytes

**DOI:** 10.3390/biomedicines10123015

**Published:** 2022-11-23

**Authors:** Sherri L. Surman, Jeremy Crawford, Pradyot Dash, Susan L. Tonkonogy, Paul G. Thomas, Julia L. Hurwitz

**Affiliations:** 1 Department of Infectious Diseases, St. Jude Children’s Research Hospital, 262 Danny Thomas Place, Memphis, TN 38105, USA; 2 Department of Immunology, St. Jude Children’s Research Hospital, 262 Danny Thomas Place, Memphis, TN 38105, USA; 3 Lentigen, a Miltenyi Biotec Company, Gaithersburg, MD 20878, USA; 4 College of Veterinary Medicine, North Carolina State University, Raleigh, NC 27607, USA; 5 Department of Microbiology, Immunology and Biochemistry, University of Tennessee Health Science Center, Memphis, TN 38163, USA

**Keywords:** *E. coli*, germ-free, gnotobiotic, T cell receptor repertoire, thymus, CD4+CD8+ T cell

## Abstract

The microbiome shapes the mature T cell receptor (TCR) repertoire and thereby influences pathogen control. To investigate microbiome influences on T cells at an earlier, immature stage, we compared single-cell TCR transcript sequences between CD4+CD8+ (double-positive) thymocytes from gnotobiotic [*E. coli* mono-associated (Ec)] and germ-free (GF) mice. Identical TCRβ transcripts (termed repeat, REP) were more often shared between cells of individual Ec mice compared to GF mice (Fishers Exact test, *p* < 0.0001). Among Ec REPs, a cluster of Vβ genes (Vβ12-1, 12-2, 13-1, and 13-2, termed 12-13) was well represented, whereas 12-13 sequences were not detected among GF REPs (Fishers Exact test, *p* = 0.046). Vα genes located in the distal region of the TCRα locus were more frequently expressed in Ec mice compared to GF mice, both among REPs and total sequences (Fishers Exact test, *p* = 0.009). Results illustrate how gut bacteria shape the TCR repertoire, not simply among mature T cells, but among immature CD4+CD8+ thymocytes.

## 1. Introduction

T cell receptor (TCR) gene rearrangement and expression patterns are non-random and depend on the environment of the developing T cell. Born et. al. first identified non-random TCR Jβ gene rearrangement events [1], and Thompson et. al. identified non-random TCRα rearrangements that changed during mouse T cell ontogeny [2]. V-Jα rearrangement patterns were biased by gene location, progressing from proximal to distal regions in the locus with mouse age. Wilson et. al. found that Vβ patterns were biased among immature thymocytes toward the expression of genes within a cluster (here termed ’12-13′, inclusive of Vβ12-1, 13-1, 12-2, and 13-2 genes, International Immunogenetics [IMGT] nomenclature) [3,4].

The diversity of a TCR repertoire will influence an individual’s overall health, particularly when the repertoire is limited and hosts are rendered vulnerable to infectious diseases. As examples, the clinical severity of tuberculosis is correlated with restricted TCR diversity, as is the escape of hepatitis C virus from cytotoxic T cell surveillance [5,6]. A skewed TCR repertoire can also render individuals vulnerable to auto-immune disease and/or to inflammatory responses caused by bacterial or viral superantigens [7,8].

The many factors that influence TCR and immunoglobulin gene rearrangement and expression patterns are not fully understood. Nucleosome structures vary along TCR and immunoglobulin loci, affecting enzyme recruitment and function. Transcription factors, including nuclear receptors, bind regulatory regions that influence DNA looping and enzyme accessibility during DNA rearrangement events [9,10,11]. Cytokines/chemokines also influence gene rearrangements, T cell differentiation/activation, and TCR repertoire development [12,13].

Bacteria modulate the peripheral TCR repertoire in blood and mucosal tissues, in part by the binding of superantigens or peptides presented by major histocompatibility complex (MHC) proteins to a subset of variable elements on TCR membrane proteins, leading to biased T cell survival [14,15]. Bacteria will further alter cytokine/chemokine patterns [16].

While the microbiome’s influences on mature T cells in the blood and gut have been well described, few studies have investigated if and how the microbiome affects TCR expression patterns among immature T cell populations. To address this topic, we examined *E. coli* mono-associated (Ec) mice and germfree (GF) mice and asked if TCR transcripts differed between mice among CD4+CD8+ (double-positive) thymocytes, immature T cells that often expresses a TCRβ membrane protein while rearranging TCRα genes [17]. Here we show that CD4+CD8+ T cells in Ec mice exhibit different TCR gene expression patterns compared to GF mice. Results identify: (i) an early stage of T cell development that is vulnerable to TCR repertoire manipulation by the microbiome, and (ii) a cell population that may be targeted in future studies to ensure TCR repertoire adequacy.

## 2. Materials and Methods

### 2.1. Mice

GF 129S6SvEv mice were derived under GF conditions by hysterectomy at the Gnotobiotic Laboratory, University of Wisconsin, Madison and then bred and housed in the Gnotobiotic Core at the College of Veterinary Medicine, North Carolina State University. To produce Ec mice, adult GF males and females were colonized with *E. coli* NC101 [18], a non-pathogenic *E. coli* isolated from stool of a normal 129S6SvEv mouse housed in specific pathogen free conditions, by oral and rectal swabbing and then paired as breeders. Thus, the offspring used in our experiments were naturally colonized during parturition and suckling. 

Male offspring were examined to avoid influences of sex on TCR sequences or cytokine patterns. Males were euthanized at 6–10 weeks of age, and thymuses and sera were collected. Intact thymuses were placed directly into sterile RMPI 1640 medium containing gentamicin plus 20% heat-inactivated fetal calf serum and shipped overnight together with sera to St. Jude Children’s Research Hospital. Vertebrate animal use was approved by the Institutional Animal Care and Use Committee at North Carolina State University. 

### 2.2. Single-Cell Sorting 

Upon receipt of thymuses after overnight shipments, single-cell suspensions of individual thymuses were prepared and strained through 70 μM strainers. Cells were stained with fluorochrome-labeled anti-CD4 and anti-CD8 antibodies (Pharmingen, BD Biosciences, Franklin Lakes, NJ, USA; antibodies were diluted 1:100 in stain wash buffer) for 20 min at 4 degrees C. Cells were washed with stain wash buffer and single cells were sorted into 384-well plates (Eppendorf twin.tec 384-well PCR plate) using a MoFlo flow cytometer (Cytomation, Fort Collins, CO, USA) fitted with a Cyclone single-cell deposition unit. In each of three experiments with 2–4 mice per group (yielding a total of 8 and 10 Ec and GF mice, respectively), thymuses were individually harvested, stained, and single-cell sorted. Plates were sealed and stored frozen at −80 °C prior to further processing. 

### 2.3. Single-Cell Sequencing of TCR

Methods for TCR sequencing of single cells have been described previously [19,20]. Briefly, cDNA synthesis was performed directly from CD4+CD8+ single cells using either an iScript cDNA Synthesis Kit (170-8891 Biorad, Hercules, CA, USA) or a Superscript VILO cDNA Synthesis Kit (117540-50 Invitrogen, Waltham, MA, USA). Multiplex, nested polymerase chain reaction (PCR) was performed with a Taq polymerase-based PCR kit (Qiagen, Germantown, MD, USA) to amplify the complementarity-determining region 3 (CDR3) of TCRα and TCRβ transcripts in each cell. A first round of PCR used a cocktail of TCR Vα gene (TRAV) forward primers, a single TCR Cα gene (TRAC) reverse primer, a cocktail of TCR Vβ gene (TRBV) forward primers, and a single TCR Cβ gene (TRBC) reverse primer. The second round of PCR used the product from the first PCR as template and a mixture of TRAV and TRBV internal primers (described previously [19]). PCR products were purified. Next generation sequencing was performed (Hartwell Center, St. Jude Children’s Research Hospital) with Sanger sequencing used for spot-check validation. 

CDR3 sequence analyses allowed identification of Vα and Vβ sequences in individual cells and the mapping of genes within the TCR genome using the IMGT database and nomenclature. It should be noted that V gene numbering systems have changed since first reports [3]. We further note that 129 mouse substrains, including the 129S6SvEv substrain used here, have diverged during historical mouse back-crossing and have not been fully characterized [3]. 

There were approximately 250 TCRβ CDR3 sequences obtained per mouse with more than 900 sequences in each of the three experiments and more than 4500 sequences overall. There were approximately 100 TCRα CDR3 sequences obtained per mouse with more than 400 sequences per experiment and more than 1900 sequences overall. Custom Python scripts and GraphPad Prism software (Version 9, GraphPad Prism, San Diego, CA, USA) were used to analyze and display data. Fishers Exact tests were used to compare groups.

### 2.4. Cytokine Analyses

The Milliplex MAP Kit (MCYTOMA-70K-PX32, Millipore, Burlington, MA, USA) was used to measure 32 distinct cytokines/chemokines in sera per manufacturer’s instructions. Two experiments were performed independently with 4–5 mice per group. Samples were read on a Luminex 200 Multiplexing Instrument (Luminex, Austin, TX, USA) using xPonent software followed by data processing with Milliplex Analyst software and GraphPad Prism Software (Version 9, San Diego, CA, USA). Unpaired *t*-test with Welch corrections and the Holm–Sidak adjustment for multiple comparisons were performed.

## 3. Results 

Thymocytes from Ec and GF mice were collected and sorted for single-cell sequencing of CD4+CD8+ T cells in three independent experiments with 2–4 mice per group. Approximately 250 TCRβ sequences and approximately 100 TCRα sequences were characterized per mouse, a ratio reflective of the CD4+CD8+ thymocyte population wherein TCR β, but not TCR α gene rearrangements are usually complete. 

**Figure 1:**
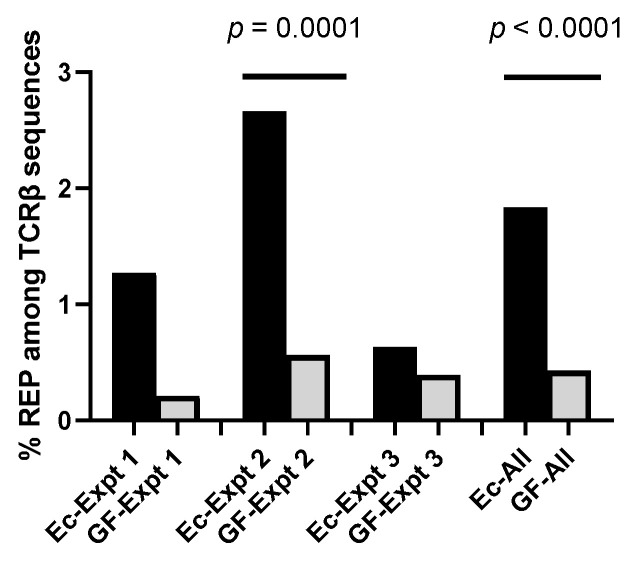
Percentages of REP sequences among all unique TCRβ sequences in Ec and GF mice. Percentages are shown for the numbers of REP sequences in Ec and GF mice in three independent experiments examined individually and together (All). There were 2 Ec and 2 GF mice in experiment 1, 4 Ec and 4 GF mice in experiment 2, and 2 Ec and 4 GF mice in experiment 3. In each experiment, REP frequencies were higher in Ec mice compared to GF mice. REP frequencies were significantly different between mouse groups in experiment 2 (the largest experiment), and in sequences from combined experiments. Fishers Exact tests were used.

To score favored sequences, we first asked if identical CDR3 TCRβ sequences were observed in more than one cell per mouse. The frequencies of repeated (REP) sequences are shown in Figure 1. As demonstrated, REPs were more common in Ec mice compared to GF mice in each of three independent experiments. Significant differences were observed in the largest experiment (Expt 2, Fishers Exact test *p* = 0.0001), and among sequences from all experiments combined (Ec-All compared to GF-All, Fishers Exact test, *p* < 0.0001)

**Figure 2:**
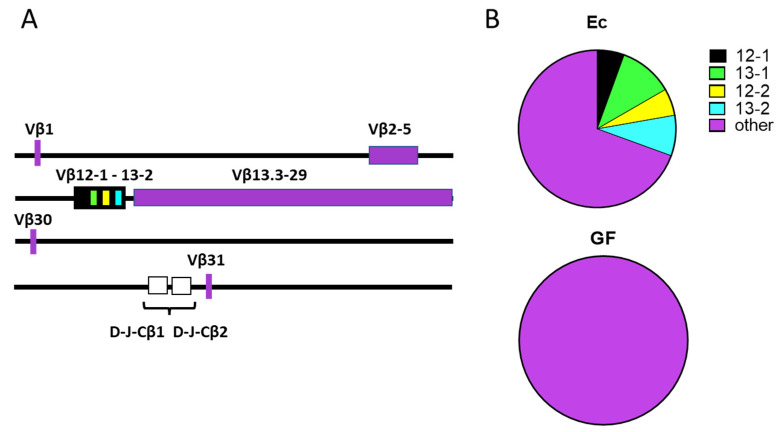
TCR Vβ 12-13 usage among REP sequences in Ec and GF mice. (**A**) Vβ gene positions in the murine TCRβ loci. Maps were based on information from the IMGT website [3]. Vβ genes in the 12-13 cluster are color-coded (black, green, yellow, blue) and the remaining genes are shown in purple. Dβ, Jβ, and Cβ genes are shown in white. (**B**) The frequencies of Vβ 12-13 sequences within REP sequences among Ec (n = 36 total sequences) and GF (n = 11 total sequences) mice are shown with color-coding. ‘Other’ refers to all REP Vβ sequences outside of the 12-13 cluster (purple).

We asked if genes from the 12-13 Vβ cluster, preferentially expressed among immature thymocytes [4], were expressed among Ec and GF REP sequences. In Figure 2A is shown the location of the Vβ 12-13 cluster in the TCRβ locus [3]. In Figure 2B are shown the frequencies of 12-13 sequences among REP sequences. As shown, the 12-13 cluster accounted for 31% of all REP sequences in Ec mice. 12-13 gene sequences were present among Ec REP sequences in each of the three independent experiments. In contrast, the 12-13 cluster was never observed among REP sequences in GF animals (Fishers Exact test, *p* = 0.046).

**Figure 3:**
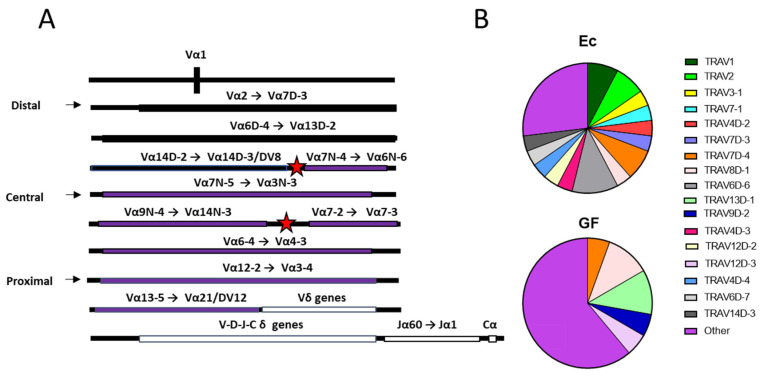
TCR distal Vα usage among REP sequences in Ec and GF mice. (**A**) Relative gene fragment positions are shown. Maps were based on information from the IMGT website [3]. Three sections of genes (distal, central, proximal) are separated by red stars. Distal Vα gene positions are shown in black, while central and proximal gene positions are shown in purple. TCR δ genes and Jα/Cα genes are shown in white (**B**) Distal Vα sequence usage among REP sequences in Ec and GF mice from the three combined experiments are shown. There were 26 TCRα REP sequences in Ec mice and 18 TCRα REP sequences in GF mice. Distal Vα sequences are named and color-coded. Central and proximal sequences are termed ‘other’ and shown in purple. Analyses of all REP sequences from the three experiments combined showed significantly more distal Vα usage in Ec mice compared to GF mice among REPs (*p* = 0.032). When unique TCR Vα sequences were examined, there was again a significantly higher frequency of distal Vα sequences in Ec mice compared to GF mice. The two largest of the three experiments (experiments 2 and 3) exhibited significant differences when analyzed independently (*p* = 0.02 for each). The heightened distal Vα usage in Ec mice compared to GF mice was most apparent when sequences from the three independent experiments were combined (n > 1900, *p* = 0.009).

As shown in Figure 3, an analysis of TCR Vα genes was also performed. There are three sets of TCR Vα genes, a consequence of evolutionary duplication [3]. In Figure 3A, these sets are labeled distal, central, and proximal, and are separated by red stars. In Figure 3B is shown the distribution of Vα sequence usage in Ec and GF mice among REP sequences. Distal Vα sequences were individually named and color-coded. As shown, distal Vα genes were expressed more frequently among REP Vα sequences in Ec mice (73%) compared to GF mice (39%, Fishers Exact test, *p* = 0.032). When total unique sequences rather than REP sequences were examined, the Ec preference for distal Vα gene usage remained. The two largest of the three independent experiments (experiments 2 and 3) exhibited significant differences in this regard (*p* = 0.02 for each). When the >1900 unique Vα sequences from three experiments were combined, the significantly higher frequencies of distal Vα gene usage in Ec mice compared to GF mice was most apparent (Fishers Exact test, *p* = 0.009).

**Figure 4:**
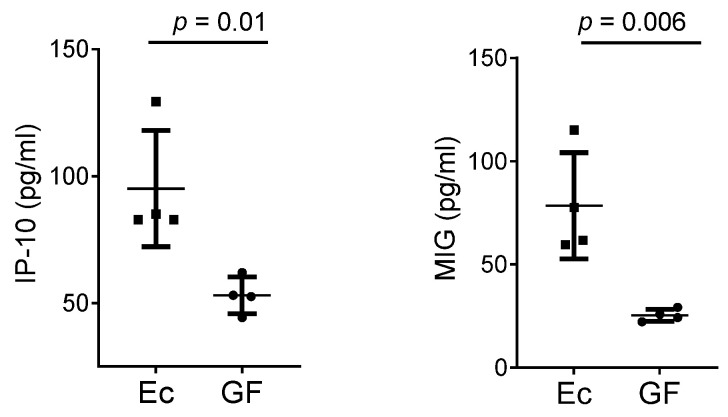
Serum chemokine levels differ between Ec and GF mice. Results are shown for IP-10 and MIG concentrations in sera of Ec and GF mice from one of two independent experiments. *p* values are shown from unpaired *t*-test. Results in a repeat experiment trended similarly. When data from the two experiments were combined, unpaired *t*-test with a Welch correction and Holm–Sidak adjustment for multiple comparisons revealed a significant difference for IP-10 (*p* = 0.042).

Because cytokines/chemokines have been shown to bias TCR repertoires in fetal thymic organ cultures [13], we asked if cytokines/chemokines differed between Ec and GF mice. As shown in Figure 4, among a panel of 32 measured serum factors, we found differences in two chemokines, each induced by interferon γ (IFNγ). These were the IFNγ-induced protein 10 (IP-10, CXCL10) and monokine induced by γ interferon (MIG, CXCL9). Trends were similar among the two independent cytokine/chemokine experiments, each using sera from 4–5 mice per group. When combined data were examined using unpaired *t*-test with a Welch correction and a Holm–Sidak adjustment for multiple comparisons, differences were significant for IP-10 (*p* = 0.042).

## 4. Discussion

### 4.1. The Microbiome Influences TCR Repertoires among Developing CD4+CD8+ Thymocytes

In this study, we asked if patterns of TCR gene expression among CD4+CD8+ thymocytes differed between Ec mice and GF mice. We found that Ec mouse CD4+CD8+ thymocytes had more frequent REP sequences than GF mice, a first indication of TCR bias (*p* < 0.0001). Among REPs, Ec mouse thymocytes expressed the Vβ 12-13 cluster and distal Vα gene fragments more often than GF mice. The Vα usage bias was additionally evident among total unique Vα sequences (*p* = 0.009). Two serum chemokines differed between GF and Ec animals, providing a potential explanation for the significantly different TCR repertoire patterns. Altogether, these data demonstrated how a single change in the microbiome could instruct a TCR repertoire bias even before T cells matured and exited the thymus.

### 4.2. Mechanisms Pertinent to TCR Repertoire Manipulation

TCR skewing in Ec mice compared to GF mice was likely influenced by a combination of mechanisms [12,13,14,15,16,21,22,23]. As a first consideration, *E coli* mono-association may have caused biased triggering of membrane TCR among developing T cells, with consequent T cell proliferation or apoptosis. The frequent presence of REP sequences in Ec mice was consistent with the hypothesis that cells bearing certain membrane TCR Vβ proteins were triggered to proliferate. Studies of blood and intestinal T cells have demonstrated how bacterial superantigens or peptides presented by MHC proteins can preferentially bind TCRs based on Vβ sequences to alter T cell survival. The Vβ 12-13 sequences (IMGT nomenclature) that were frequent among REP sequences in Ec mice encode known peripheral T cell targets of bacterial superantigens [15,23]. As a second non-mutually exclusive consideration, we propose that the microbiome can influence the frequencies and positions of TCR gene rearrangements during CD4+CD8+ thymocyte development. CD4+CD8+ thymocytes have often completed TCRβ gene rearrangements and are engaged in TCRα gene rearrangements. If immature T cells are triggered to increase successive TCRα gene rearrangement frequencies in response to *E coli*-mono-association, they will progressively delete proximal Vα genes and utilize more distal Vα genes in rearrangement events.

Other potential influences on TCR rearrangement/expression by the microbiome may include the release of fatty acids that regulate nuclear receptors (e.g., peroxisome proliferator-activated receptors, PPARs) to modify gene expression [24]. Bacteria can additionally influence Aire expression [25], and intestinal microbes can promote migration of dendritic cells from the colon to the thymus to perturb thymus distribution of PLZF (a BTB-zinc finger protein)-expressing innate lymphocytes [26]. Peripheral peptides can cause apoptosis among CD4+CD8+TCRlo thymocytes [27]. These processes likely function in unison and may each be modified by *E coli*-mono-association to influence TCR repertoire development in thymic tissues.

### 4.3. Cytokines and Chemokines

Cytokines/chemokines such as IFNγ are associated with gut microbial metabolic pathways and may alter TCR gene rearrangement, T cell development, and T cell survival [16]. In Figure 4 we showed that serum IP-10 and MIG were increased in Ec mice compared to GF mice. These two factors are structurally and functionally related, each induced by IFNγ, and each able to bind CXCR3, a receptor known to characterize T cells, including a small subset of CD4+CD8+ thymocytes [28]. IP-10 and MIG are produced by thymic epithelial cells and can attract cells including CD8+ single-positive T cells, γδ T cells and NK cells [29]. We therefore consider that IP-10 and MIG in mice maintained with a normal commensal microorganism (Ec) may have modulated TCR profiles in CD4+CD8+ thymocytes.

### 4.4. Clinical Implications of a TCR Bias

TCR repertoire breadth is critical for control of infectious diseases. A restricted repertoire, as is often observed in aged populations, will leave holes in the immune response, rendering individuals vulnerable to infectious diseases. A skewed repertoire can also render an individual susceptible to auto-immune disease and/or to super-antigen induced inflammation causing toxic shock, a life-threatening disease [30]. By further investigating the influence of microbiota on the TCR repertoire, with attention to the thymus, scientists may learn to predict and prevent limitations in TCR profiles, and thereby prevent disease vulnerabilities.

## 5. Conclusions

We show that *E. coli* mono-association is sufficient to induce TCR bias among immature T cells in the thymus. In Ec mice compared to GF mice, there were higher frequencies of REP sequences, higher frequencies of Vβ 12-13 gene usage among REPs, and higher frequencies of distal Vα gene usage. Our results show that the microbiome can shape TCR repertoires, not simply among mature T cells, but among immature CD4+CD8+ thymocytes. Results highlight (i) an early stage of T cell repertoire development that is vulnerable to influences of the microbiome, and (ii) a thymocyte population that may now be targeted to ensure TCR repertoire adequacy.

## Data Availability

Data are available upon request to the corresponding author.

## Abbreviations

TCR	T cell receptor
V	Variable
D	Diversity
J	Joining
C	Constant
GF	germ-free
Ec	*E. coli* mono-associated
REP	repeat
